# Obtaining an Equiaxed Ultrafine-Grained State of the Longlength Bulk Zirconium Alloy Bars by Extralarge Shear Deformations with a Vortex Metal Flow

**DOI:** 10.3390/ma16031062

**Published:** 2023-01-25

**Authors:** Alexandr Arbuz, Anna Kawalek, Kirill Ozhmegov, Evgeniy Panin, Medet Magzhanov, Nikita Lutchenko, Vasily Yurchenko

**Affiliations:** 1 Core Facilities Department, AEO Nazarbayev University, 53 Kabanbay Batyr Ave., Astana 010000, Kazakhstan; 2 Metal Forming Department, Częstochowa University of Technology, ul. J.H. Dąbrowskiego 69, 42-201 Częstochowa, Poland; 3 Metal Forming Department, Karaganda Industrial University, 30 Republic Ave, Temirtau 101400, Kazakhstan; 4 Mechanical Engineering Department, Abylkas Saginov Karaganda Technical University, 56 Nursultan Nazarbayev Ave, Karaganda 100027, Kazakhstan

**Keywords:** radial shear rolling, ultrafine-grained structure, zirconium alloy, gradient structure, severe plastic deformation, large deformations, FEM simulation

## Abstract

The method of radial shear rolling makes it possible to achieve comparable to high pressure torsion (HPT) method ultrahigh degrees of total strain level in combination with the vortex metal flow character for long-length large bulk bars unable by HPT and many other processes of sever plastic deformation (SPD). Sequential rolling of the Zr-1%Nb alloy was carried out under extreme conditions on two radial shear rolling mills with a total diameter reduction ε = 185% and a maximum total strain level = 46 mm/mm. The strain level and its cross-section distribution assessment by finite element method (FEM) simulation was studied. The final bar cross-section structure type distribution detailed study 1 mm resolution by electron back scatter diffraction (EBSD) mapping was performed. A gradient structure with a predominance of the equiaxed ultrafine-grained (UFG) state was found. The deformation level rising did not allow to refine it in the periphery zone more than that obtained nearly middle of the processing, but it allows for significant change in the axial zone structure. The additional large warm deformations by radial shear rolling have no additional grain refinement effect for already 300–600 nm refined zone. An equiaxed UFG structure was obtained in a relatively large volume of the sample with a reduced gradient towards the non-UFG center zone in regard to known works.

## 1. Introduction

Zirconium-based alloys are one of the main materials of nuclear engineering and are used for thermal neutron reactors such as a pressurized water reactor (PWR) or water-water energetic reactor (WWER) fuel elements (fuel rods) shells and plugs manufacturing [1]. The relatively low zirconium structure is damaged by a neutron flux, to move the main material engineering focus for study the damage effects by uranium nucleus fission fragments, which is severely influenced to the structural elements’ durability. The protecting of fuel rods shell surface and its mechanical properties improving is one of the most urgent problems of modern nuclear power engineering.

It is possible to dramatically improve the mechanical properties of a metal by transforming its structure into an ultrafine-grained and nanostructural state [2,3]. There are also plenty of grain boundaries in the material volume, which will function as a drain surface for continuously formed radiation defects. As a result, the radiation resistance of the vessel structural parts will increase [4,5].

The ultrafine-grained structure (UFG) forming can be achieved by the next conditions together. The metal must be subjected to large values of severe plastic deformation (ε > 6–8) with the all-round compression condition of 1 GPa or higher using the low temperature processing mode [3,6]. The metal flow nonmonotonicity will also play a significant role here. The UFG forming conditions implementation is easy for the discrete processes such as pressing (equal channel angular pressing—ECAP) [7,8,9], forging [10], and high-pressure torsion (HPT) [6,11], but production complications solving is relevant for the long-length products with inevitable tensile stresses, such as rolling [12] or drawing [13]. The best improvement effect can be given by the vortex metal flow implementation under the all-round hydrostatic compression conditions [14]. This scheme is achievable for the long-length products obtaining by the radial shear rolling method [15,16]. The method will make it possible to achieve very large deformations due to the trajectory velocity shift of metal layers relative to each other by the rod cross-section [17]. The most detailed mechanics of the process in source [18] is described. This method of UFG structure obtaining is not as widely studied as the already referred to ECAP, HPT, or some other kinds of forging, and therefore various specific and limiting cases of radial shear rolling process study have some scientific interest. It is especially relevant for various special materials processing, including rare materials.

The radial shear rolling method has become in recent years increasingly popular for significant structure refinement of steel [16,19,20,21,22,23,24], titanium [25,26], aluminum and magnesium alloys [27,28,29], copper [16], zirconium [30], and other alloys [31,32,33,34,35]. However, in many cases, the deformation on the one setup is used, and the total deformation by diameter does not exceed ε = 50%. In this case, significant total strain up to 35 mm/mm in the peripheral parts of the sample was performed and a gradient structure was formed with an equiaxed UFG periphery and an elongated texture in the axial part.

The main purpose of this work is the experimental implementation of significantly larger total strains than in previously known works regarding the method of radial shear rolling and its effect on the Zr-1%Nb alloy example structure and properties changes study.

## 2. Methodology, Materials and Equipment

**Figure 1:**
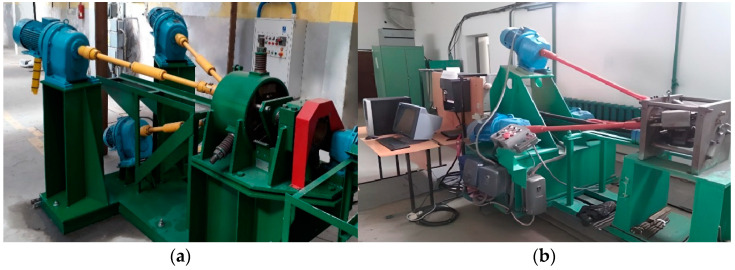
The used radial shear rolling mills (**a**) RSP-14/40 (PCz, Poland); (**b**) RSP-10/30 (KSU, Kazakhstan).

For the large deformation level achievement, the warm rolling from a diameter of 37 mm to a diameter of 13 mm on two radial shear rolling mills with different characteristics was used. These dimension values are the limit sizes for the incoming bar for the larger mill and the outgoing bar for the smaller mill. In the work, the mills RSP-14/40 (larger) at the Czestochowa Polytechnic University (Poland) and RSP-10/30 (smaller) at the Karaganda Industrial University (Kazakhstan) were used. The ranges of its operational diameters overlap. The first rolling mill actually provides deformation from 37 mm to 20 mm, and the second mill from 30 mm to 13 mm. The used rolling mills are shown in Figure 1.

The samples’ heating and temperature control on the Nabertherm R170/750/12 tube furnace were carried out. The rolling process stress-strain state assessment and the final total strain level after all rolling processing route by two mills using the numerical simulation by the finite element method (FEM) were performed using the Deform-3D (SFTC) software package.

Electron microscopy methods were used to study microstructural changes. Maps of the crystallographic orientation of grains and the grain structure as a whole were taken with electron backscatter diffraction (EBSD) on a high-resolution scanning electron microscope (SEM) Crossbeam-540 (Carl Zeiss, Oberkochen, Germany) with a NordlysNano EBSD attachment (Oxford Instruments, Abingdon, UK). Diffraction pattern recognition and mapping was performed with HKL Channel-5 Tango software (Oxford Instruments, Abingdon, UK). The fine structure was studied on a JEM-1400PLUS Transmission Electron Microscope (TEM) (JEOL, Tokyo, Japan) in bright field (BF) and selected area diffraction (SAED) modes.

Sample preparation of zirconium samples for both methods of electron microscopy was carried out by electrolytic polishing on Struers equipment: TenuPol-5 for TEM and LectroPol-5 for SEM/EBSD. In both cases, A3 electrolytes recommended by the manufacturer were used (perchloric acid—78 mL, distilled water 90 mL, ethanol 730 mL, and butoxyethanol 100 mL). In both cases, forced cooling of the electrolyte was used with the help of additionally built-in Julabo 600F cryostats in the Struers setups. Cooling of the electrolyte is usually not separately described in sample preparation protocols, but it has a very strong positive effect, especially for the TEM sample. Based on the works [36,37], our own mini-R&D was carried out with the search for the optimal combination of electrolyte temperature, voltage, and flow rate of the electrolyte surrounding the sample. By varying the indicated parameters, studying the type of current-voltage characteristic and the quality of the resulting sample, the following optimal values were established. For TEM-sample: temperature—−20 °C; voltage—37V; and flow rate—32. For EBSD/SEM sample: temperature—+5 °C; 30 s, voltage—10V, flow rate—13 for polishing stage; and 40 s, voltage—8V, flow rate—13 for etching. These protocols, as well as work [36], can be recommended for the preparation of samples from a heavily deformed, hardened, and saturated dislocations alloy M5 and Zr-1%Nb.

Sample cutting for all types of subsequent sample preparation was performed on a precision cutting machine Brilliant−220 (QATM) with intensive water cooling and a cutting speed of 5 μm/s to minimize deformation-temperature damage to the structure. Coarse-grained cut-off wheels were used at 700RPM. For electrolytic polishing of TEM samples, longitudinal plates 0.3 mm thick were cut from the central part of the rod, and 3 mm disks were punched out of them with the Disc Punch tool by Gatan. For SEM/EBSD, thicker plates 2 mm thick were also cut from there. The cutting scheme is shown in detail in the experimental part.

## 3. Numerical Simulation

The main goal of computer simulation was to estimate how much the level of accumulated strain increases during sequential warm rolling of a zirconium alloy in 2 radial shear rolling mills under technically limiting conditions. Knowledge of the limiting available values and the distribution of the level of accumulated strain over the cross-section of the bar is necessary to study the effect of very large shear strains with a vortex flow of metal over layers on changes in the structure and properties of the metal, which is the main goal of the work. This is the reference for interpreting the resulting structure.

Since the Zr-1%Nb material is very specific, its rheological properties necessary for creating a computer model are not available in the databases of popular FEM modeling packages. The work on obtaining a rheological database was carried out jointly with prof. H. Dya, prof. A. Kawalek, and Dr. K. Ozhmegov in Poland at the Czestochowa University of Technology and available for in the range of temperatures 20–650 °C and strain rates 0.5–15 s^−1^ are available online at [38].

**Figure 2:**
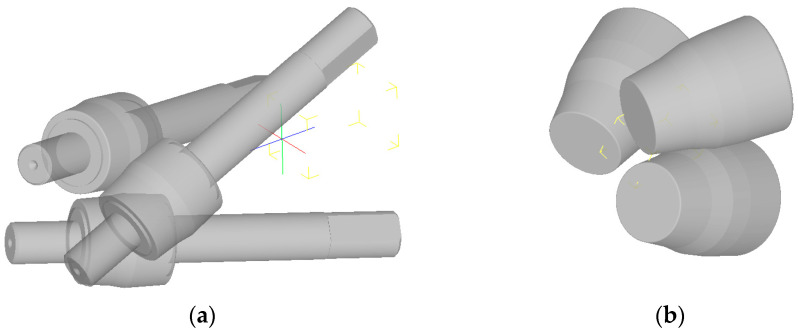
Models of radial shear rolling mills: (**a**) RSP-14/40 mill; (**b**) RSP-10/30 mill.

For a radial shear rolling model, the parameters of two mills were used: RSP 14/40 rolling mill (Figure 2a) at Częstochowa University of Technology for rolling from 37 mm to 20 mm and RSP-10/30 rolling mill (Figure 2b) at Karaganda Industrial University for rolling from 20 mm to 13 mm. The rolls have a different dimension and different design, but the main positions angles of the rolls’ axis are the same and the effect for metal flow peculiarities and strain-stress state should be the same, too.

The initial billet with a diameter of 37 mm and a length of 150 mm was rolled on both mills with accelerated reduction. The rolling route was as follows: 37-36.5-34-32-30-28-26-23-20 mm on the first mill and 20-17-13 mm on the second mill.

The heating temperature 530 °C for rolling was chosen, according to the results of work [30]; the rotation speed of the rolls was equal to 100 rpm. The friction coefficient at the contact of the billet and rolls was assumed to be 0.7, as the recommended value for hot rolling in deform. When rolling, the rolls were taken by rigid bodies, and the material of the billet was elastic-plastic.

To analyze the metal processing level during deformation, the parameter “equivalent strain” is usually used. Since radial shear rolling is a cross type of rolling, it is advisable to study the equivalent strain in the cross-section of the workpiece. It allows to evaluate not only the numerical values of the parameter, but also the nature of its distribution over the cross-section during deformation.

**Figure 3:**
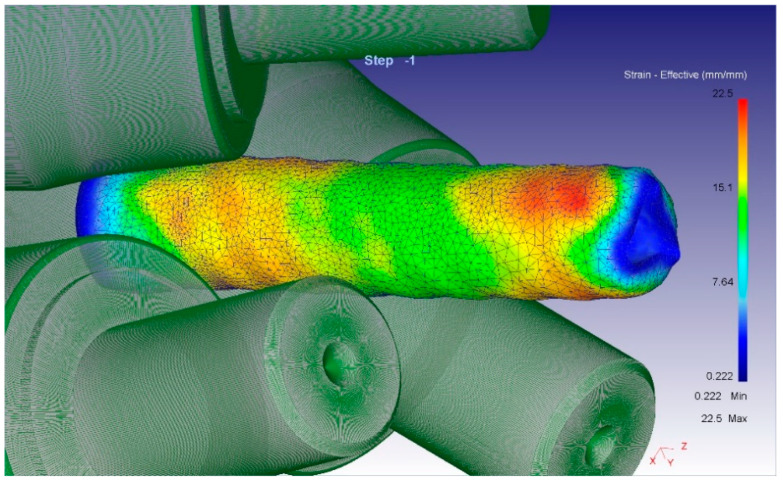
General sample view in equivalent strain colors and mesh in deformation zone after 37–30 mm of radial shear rolling at RSP-14/40 mill.

**Figure 4:**
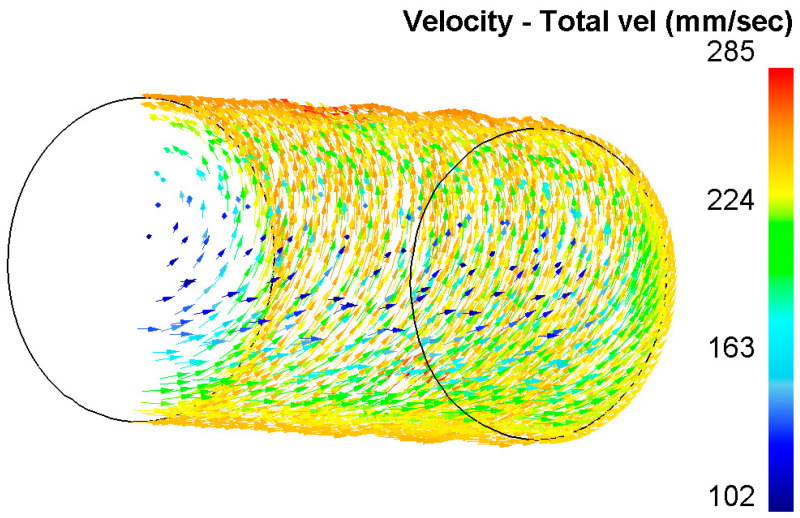
Vortex metal flows in deformation zone after 37-30 mm of radial shear rolling at RSP-14/40 mill.

When analyzing the equivalent strain (Figure 3), it was found that the distribution of this parameter has a ring type. In all cross-sections, there are clear ring zones of strain development. At the first pass, when reduction was 1.5 mm per pass, the difference of strain values between the center and surface is high: surface layers are processed up to 6.5, while central layers remain almost unprocessed (strain level 1.8 ÷ 2.0). The metal flows vectors characterization and its distribution in deformation zone after 37–30 mm radial shear rolling processing are visualized in Figure 4.

After reduction series for 2 mm (passes 3–6) in the axial zone, the strain level is smoothly grows from 6 to 9.5 approximately, while in the surface zone, where the maximum effect of shear deformation is observed, the strain level changes from 11 to 22 approximately.

When the reduction level was increased up to 3 mm per pass, this led to an increase in the strain level difference between the center and surface. After two passes (passes 7–8) in the axial zone, the strain level was 14 approximately, and in the surface zone, the strain level was 29 approximately.

After the eighth pass the billet was deformed at RSP-10/30 mill. New rolls configuration and smaller workpiece size led to intensification of shear strain formation over the cross-section. Here, after the nineth pass with a 3 mm reduction, central layers were processed up to 23 in strain level, while surface layers were processed up to 35 approximately. After the tenth pass with a 4 mm reduction, central layers were processed up to 25 in total strain level, while surface layers were processed up to 46 total strains approximately.

**Figure 5:**
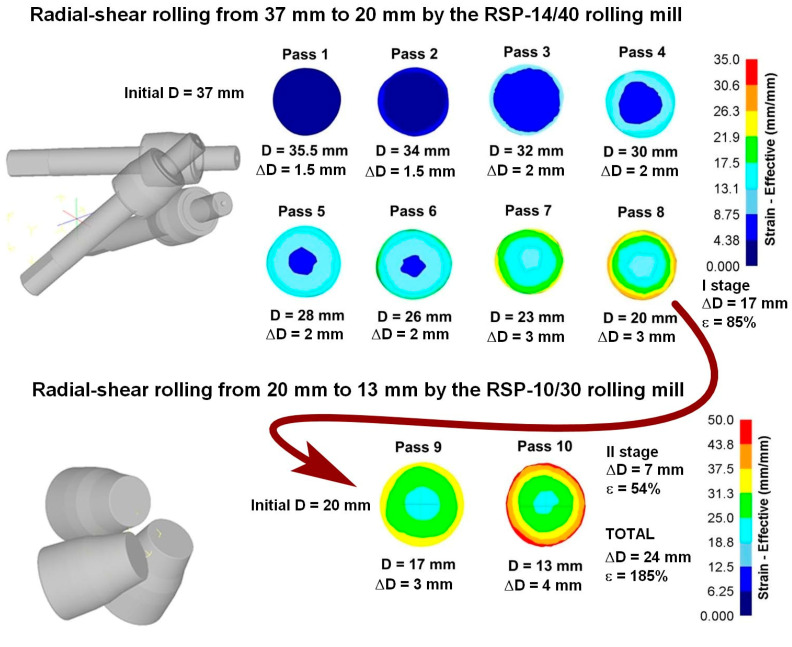
Equivalent strain through 10 passes of radial shear rolling at RSP-14/40 and RSP-10/30 rolling mills.

**Figure 6:**
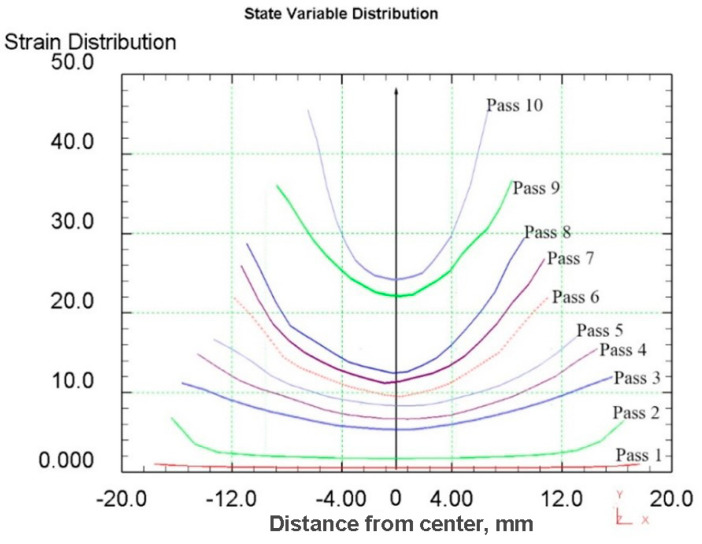
Graphs of equivalent strain distribution over the workpieces cross-section by all treatment passes.

It can be seen from the simulation that rolling on the second RSP-10/30 mill (passes 9 and 10) significantly increases the degree of accumulated deformation in the central zone, which makes it possible to expect a significant change in the structure. Equivalent strain cross-section step-by-step through all 10 passes is shown in Figure 5 and Figure 6.

## 4. Experimental

The experiment was carried out by sequential rolling along the route described in the previous section on modeling in Figure 4. The initial billet was heated for 37 min to 530 °C, then sequentially, from the one heating it was rolled 8 passes on the RSP-14/40 rolling mill to the 20 mm diameter reaching. The process of reconfiguring the mill for the next rolling size was as fast as possible and the time between the passes was no more than 1–2 min. At this time, for the heat losses prevention, the workpiece was placed back into the furnace. Rolling took place in the air, without the roll’s deformation zone cooling by water or suspension. After the final diameter was reached, the workpiece was intensively water cooled.

**Figure 7:**
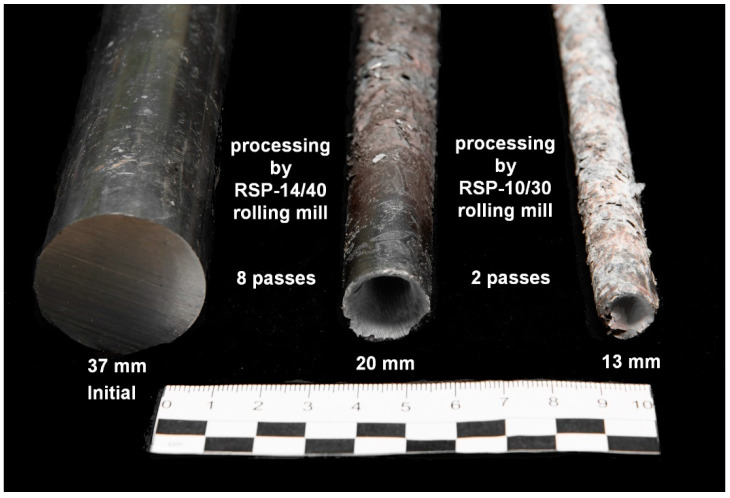
Samples of Zr-1%Nb alloy by rolling processing stages.

The obtained 20 mm workpiece had the next stage rolling on the RSP-10/30 mill. It was also carried out after its heating for 20 min to 530 °C. Rolling for a final 13 mm diameter with one heating condition in 2 passes with a maximum allowable reduction of 4 mm per pass was carried out. The rolled samples according to the rolling stages are shown in Figure 7.

All of the single rolling reductions during both treatments by numerical simulation were determined as the maximum possible values under these conditions, and a real experiment confirmed them. During several initial passes, the workpiece bar was stopped and stuck in the rolls. The hot workpiece extracting and process restarting did not take much time, but this clearly indicated the really limiting processing conditions on this equipment. Additionally, the front and rear workpiece bar ends shape changing visual comparison with computer models is very similar. This fact the accuracy of the obtained earlier Zr-1%Nb material database [38] and the adequacy of the model construction confirms.

**Figure 8:**
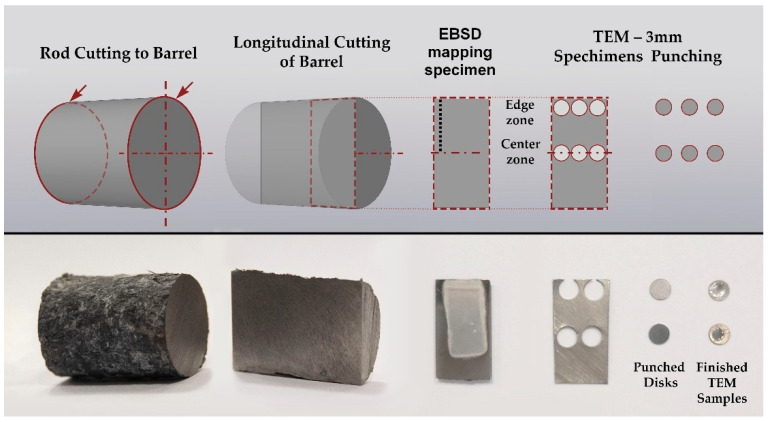
The sample cutting scheme for various characterization methods.

After the rolling processing, the sample bar according to the scheme shown in Figure 8 was cut for examination. A 20 mm length barrel is cut out of the sample bar, which is sawn in half along the axis. From both halves of the section, 2 plates were cut including one 0.3 mm thickness piece for the preparation of TEM samples to characterize the fine structure of the central zone and the peripheral zone of the sample. Another thicker sample piece was used for precision electrolytic polishing of the EBSD specimen preparation for a detailed sample cross-section structure analysis.

## 5. Results and Discussion

**Figure 9:**
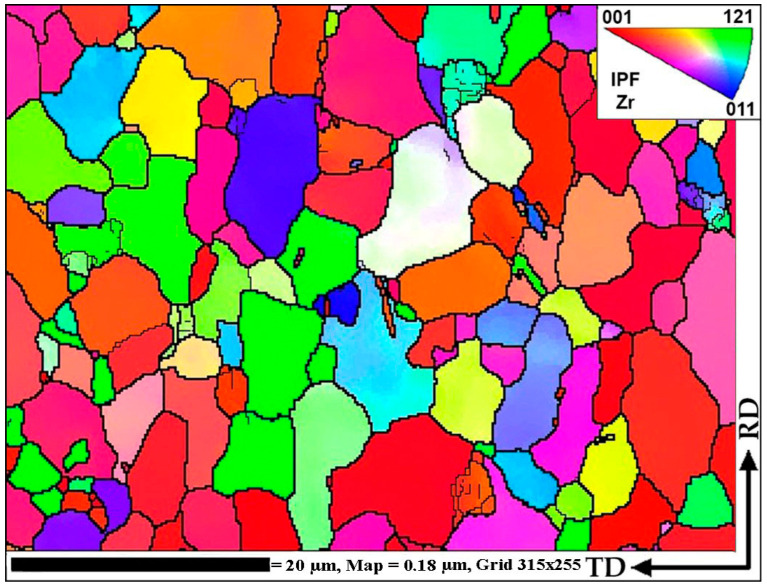
The initial sample structure characterization by EBSD map.

Based on the total strain cross-section distribution graphs by the rolling passes in Figure 5, the large difference between the central zone and the periphery is visible. Additionally, on the graph, the last two passes (9 and 10) on the RSP-10/30 mill were made to stand out noticeably. This fact makes it possible to significantly increase the degree of deformation of the central zone and change the nature of its distribution. It is known that a deformation level and its distribution is similar to that obtained in passes 1–8 which leads to the forming of a gradient structure with UFG at the periphery and a texture in the central zone [30]. A significant increase in the total strain level in the central zone can change the initial structure (Figure 9). To clarify what is happening inside the metal, a much more detailed study of the rolled bar cross-section is required, because most of the previously known radial shear rolled structure characterization works no more in three cross-section point was carried out.

EBSD was chosen as the main method for characterization the microstructure since it allows performing the most accurate positioning of the field of view to study changes in the structure over the entire cross-section of the bar with sufficient resolution. Additionally, EBSD provides crystallographic information about the orientation of the grains and automatically recognizes and allows you to analyze the structure with a set of statistics. TEM was also used, but as an auxiliary method for characterizing fine structure elements.

**Figure 10:**
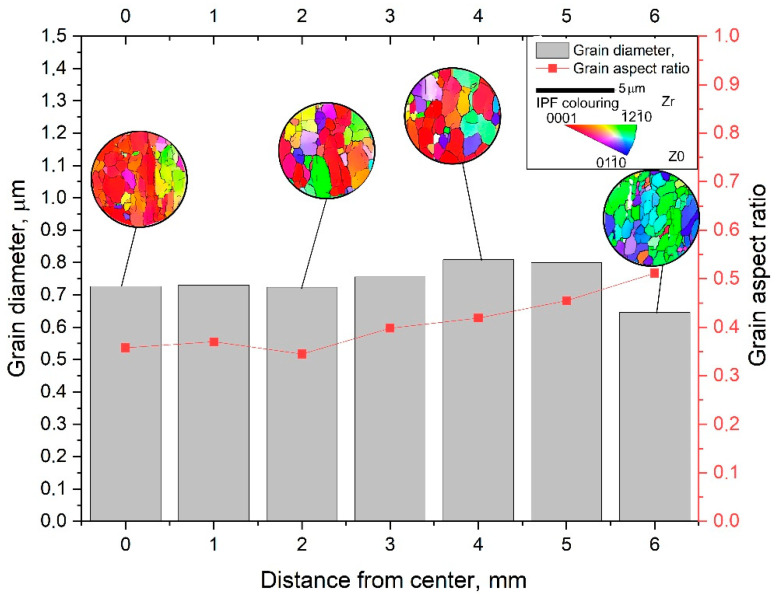
The final rolled sample cross-section structure EBSD study.

**Figure 11:**
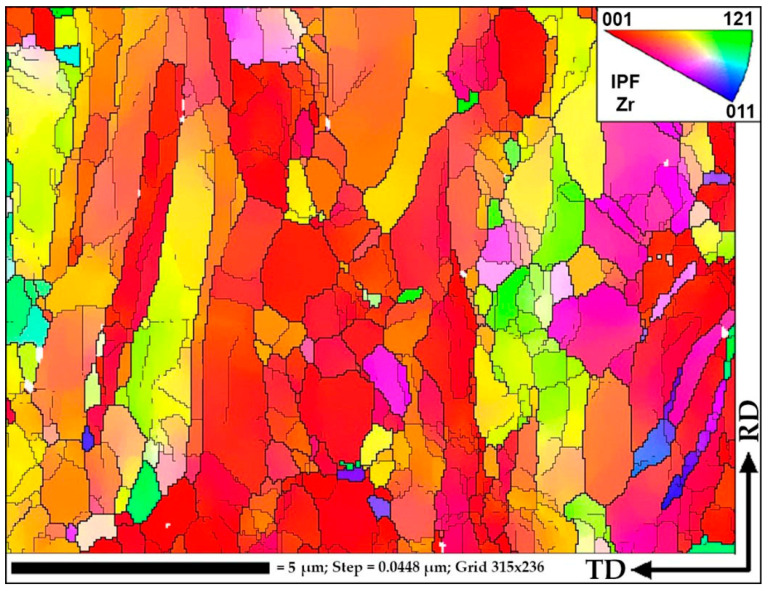
The central (axial) zone of the final rolled sample EBSD map.

**Figure 12:**
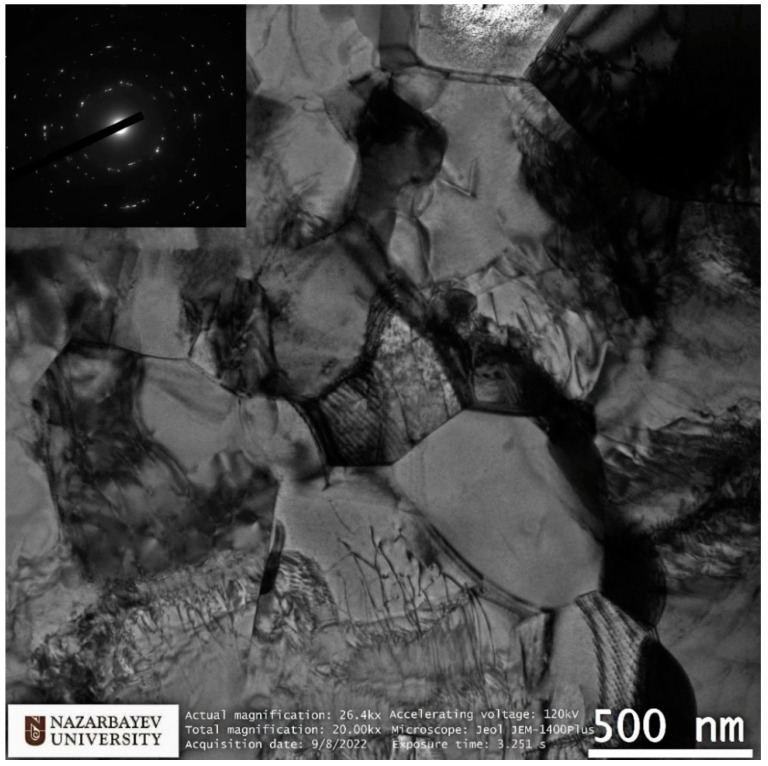
TEM-characterization of final sample peripheral structure.

**Figure 13:**
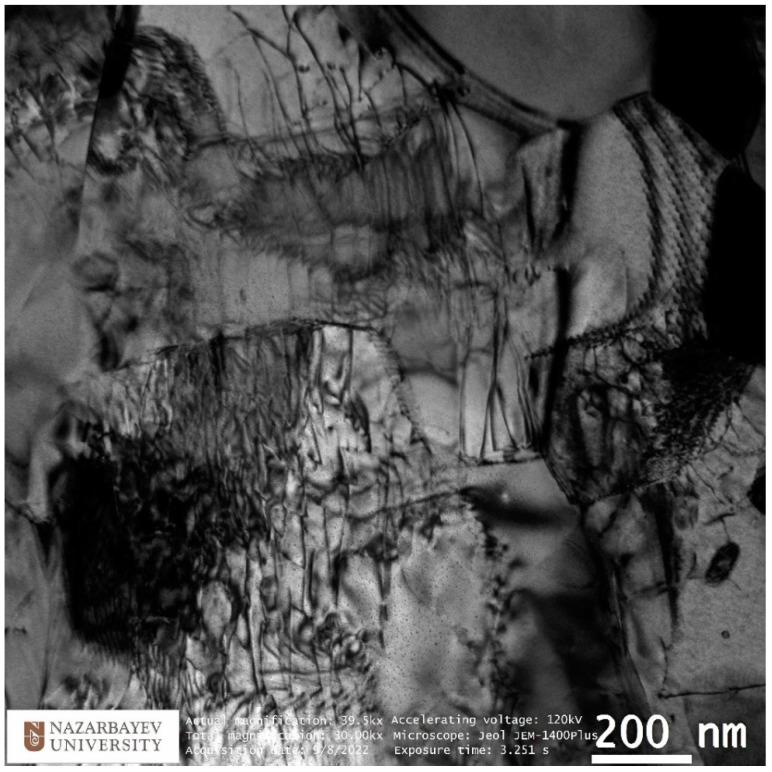
TEM-characterization of final sample peripheral fine structure peculiarities.

EBSD maps were taken along the bar radius with a 1 mm step on the longitudinal section after the final deformation according to Figure 8. The maps were statistically processed, and information was obtained on the average grain diameter and the average ratio of the largest and smallest grain size. Both are the most important numerical characteristics of the microstructure. It is impossible to use only the grain size because we are talking about a possible transformation of a gradient structure with texture elements. Each map had at least 70 measured grains; the numerical values are shown in the form of graphs in Figure 10. A typical view of the structures in the respective zones is shown in thumbnails above the graph. A certain gradient of elongation of the grains towards the center of the bar is clearly visible (decrease in the value of the aspect ratio). In this case, the average size remains approximately the same throughout the entire section with a sharp dip in the most extreme value. Additionally, there is a noticeable gradient in orientations with a clear increase in the predominance of orientations to the axis of the bar coaxially with the direction of rolling. In this case, the shape of the grains differs significantly from those described earlier in the axial zone after such a process [30], and more like a transition zone. A full size EBSD map of the axial zone is shown in Figure 11. Also, you can meet closer the original full-scale EBSD map files for all of points in supplementary materials by Figures S1–S8 using for interpretation IPF 1 coloring legend by Figure S10. A TEM study of the fine structure of the peripheral zone is shown in Figure 12 and Figure 13. You can compare those images with typical initial fine structure by TEM on the Figure S9 in supplementary materials.

As can be seen from Figure 11, in the central zone of the bar, the structure is in the process of changing under the influence of large deformations and vortex flow elements. Most likely, the texture changes by fragmentation and rotation of elements of long textural grains, as well as grain growth at some points or combination of textural ones due to the effects of dynamic recrystallization. It is clearly noticeable that the ongoing processes have a cluster characteristic. There are entire regions of grains with similar orientations that are very different from the orientations of neighboring regions. At the same time, there are very rare cases when an individual grain of a very different orientation from the environment comes across. That is, low-angle boundaries between neighboring grains and high-angle boundaries between clusters predominate. What is happening is reminiscent of one of the iterations of the process of formation of the UFG structure shown in Figure 3 in [7].

TEM study according to the position of the transparent part of the sample corresponds to an area remote from the center by about 5 mm or 1.5 mm from the edge. The study is fully consistent with the EBSD data from this and adjacent areas, as well as with data from similar studies of the peripheral region in bars obtained by radial shear rolling [16,24,28,30] and other SPD methods [7,8,9]. Here, it is important to note the equiaxed nature of grains and high-angle boundaries, as evidenced by electron diffraction in the corner of the image. It is also important to note that the grain size of the peripheral zone does not undergo significant changes during additional rolling from 20 mm to 13 mm compared to data known from sources. Additionally, the size is comparable to that achieved by the ECAP method. It is important to note the very high saturation of grains with dislocations. The fine structure of dislocations is shown in more detail in Figure 13. The initial fine structure does not contain such a large dislocation number.

The phenomena of sharp changing the grain size on the most peripheral sample cross-section point (X = 6 mm) in Figure 10 can be explained not only by the extra-large strain level, but much more by the cooling effect of rolls surface. In this experiment, the grain refinement possibility limit by deformation in the warm deformation conditions was found. The divergence can occur by the other factor local fluctuation, and one of the strongest grain refinement factors is temperature.

The purpose of this work was to study the processes of microstructure change occurring in relatively large volumetric bars under the large plastic deformations influence. Such large levels of deformation are usually achievable in the process of high-pressure torsion of small disks but are difficult to achieve in large volumetric bars, and therefore have been little studied. For this purpose, sequential processing of the Zr-1%Nb alloy was carried out on two radial shear rolling mills using all its capabilities to the maximum degree of total strain achievable.

1. Based on the FEM simulation, graphs of the total strain by the passes were built, including eight passes on one rolling mill and two passes on the other rolling mill. After the rolling processing from 37 mm to 13 mm (ε = 185%), the total strain distribution for all cases has a gradient character with a final maximum (46 mm/mm) in the peripheral zone and a minimum (25 mm/mm) in the axial zone. This strain level is associated with the predominance of shear deformations due to the vortex flow of the metal, which is clearly seen in the simulation. The use of the second rolling mill makes it possible to significantly increase the total strain level and probable structure processing.
2. After a full-scale warm rolling (530 °C) experiment regarding the FEM-simulated rolling route, the cross-section of the samples was examined by EBSD methods with a high resolution of 1 mm. The small gradient presence regarding grain size and elongation from the periphery to the center by mapping was proven.
3. The very high-quality equiaxed ultrafine-grained structure with high-angle boundaries in the peripheral sample zone was obtained. This fact was also confirmed by high-resolution TEM in addition to EBSD maps. The fine structure of the grains is very saturated with dislocations; however, the grain size corresponds to that obtained with smaller total strains level, and the grains of the rod’s periphery do not refine more than the values known from the literature sources for this and other methods.
4. The grains of the axial sample zone represent a transitional type of structure, differing significantly from that known from the sources regarding this process. Instead of a rolling texture with long elongated grains and sharp-edge boundaries, the central zone has a cluster colony of small and ultrafine grains (0.3–1 µm) with rare large grains (up to 2–3 µm). The grains within the clusters have low-angle boundaries between themselves, while the boundaries between the clusters are high angle.

## 6. Conclusions

Based on the study results, the following conclusions can be made.

- The additional large warm deformations by radial shear rolling have no additional grain refinement influence for already 300–600 nm refined zone.
- The central sample zone elongated rolling texture after the total strain significantly increased into a more equiaxed structure type that begins to transform.
- The current total strain value is insufficient to obtain a completely homogeneous cross-section structure, and the processing temperature mode is still so high, thus is the role of recrystallization and strain heating processes is also significant.
- An equiaxed UFG structure was obtained in a relatively large volume of the sample with a reduced gradient towards the non-UFG center zone regarding known works.

## Data Availability

Not applicable.

## Supplementary Materials

The following supporting information with all of used EBSD maps originals can be downloaded at: https://www.mdpi.com/article/10.3390/ma16031062/s1, Figure S1: 0 mm distance from the center, Figure S2: 1 mm distance from the center, Figure S3: 2 mm distance from the center, Figure S4: 3 mm distance from the center, Figure S5: 4 mm distance from the center, Figure S6: 5 mm distance from the center, Figure S7: 6 mm distance from the center, Figure S8: INITIAL Structure by EBSD, Figure S9: INITIAL Structure by TEM, Figure S10: EBSD legend IPF 1 colouring.

## References

[1] Zinkle S.J., Was G.S. (2013). Materials challenges in nuclear energy. Acta. Mater..

[2] Valiev R.Z., Alexandrov I.V., Zhu Y.T., Lowe T.C. (2002). Paradox of strength and ductility in metals processed bysevere plastic deformation. J. Mater. Res..

[3] Edalati K., Bachmaier A., Beloshenko V.A., Beygelzimer Y., Blank V.D., Botta W.J., Bryła K., Čížek J., Divinski S., Enikeev N.A. (2022). Nanomaterials by severe plastic deformation: Review of historical developments and recent advances. Mater. Res. Lett..

[4] Nita N., Schaeublin R., Victoria M. (2004). Impact of irradiation on the microstructure of nanocrystalline materials. J. Nucl. Mater..

[5] Etienne A., Radiguet B., Cunningham N.J., Odette G.R., Valiev R., Pareige P. (2011). Comparison of radiation-induced segregation in ultrafine-grained and conventional 316 austenitic stainless steels. Ultramicroscopy.

[6] Valiev R.Z., Islamgaliev R.K., Alexandrov I.V. (2000). Bulk nanostructured materials from severe plastic deformation. Prog. Mater. Sci..

[7] Prithivirajan S., Naik G.M., Narendranath S., Desai V. (2023). Recent progress in equal channel angular pressing of magnesium alloys starting from segal’s idea to advancements till date—A review. Int. J. Lightweight Mater. Manuf..

[8] Valiev R.Z., Langdon T.G. (2006). Principles of equal-channel angular pressing as a processing tool for grain refinement. Prog. Mater. Sci..

[9] Ciemiorek M., Chromiński W., Jasiński C., Lewandowska M. (2022). Microstructural changes and formability of AL–Mg ultrafine-grained aluminum plates processed by multi-turn ECAP and upsetting. Mater. Sci. Eng. A.

[10] Tolkushkin A.O., Lezhnev S.N., Naizabekov A.B. (2019). Development and research of the billet forging technology in the newly designed step-wedge dies. Mater. Sci. Forum..

[11] Li Z., Ding H., Huang Y., Langdon T.G. (2022). An evaluation of the mechanical properties, microstructures, and strengthening mechanisms of pure Mg processed by high-pressure torsion at different temperatures. Adv. Eng. Mater..

[12] Jiang L. (2008). Texture, Microstructure and mechanical properties of equiaxed ultrafine-grained Zr fabricated by accumulative roll bonding. Acta. Mater..

[13] Naizabekov A., Volokitina I., Volokitin A., Panin E. (2019). Structure and mechanical properties of steel in the process “pressing–drawing”. J. Mater. Eng. Perform..

[14] Shahbaz M., Pardis N., Ebrahimi R., Talebanpour B. (2011). A novel single pass severe plastic deformation technique: Vortex extrusion. Mater. Sci. Eng. A.

[15] Diez M., Kim H.-E., Serebryany V., Dobatkin S., Estrin Y. (2014). Improving the mechanical properties of pure magnesium by three-roll planetary milling. Mater. Sci. Eng. A.

[16] Naizabekov A.B., Lezhnev S.N., Dyja H., Gusseynov N., Nemkaeva R. (2017). The effect of cross rolling on the microstructure of ferrous and non-ferrous metals and alloys. Metalurgija.

[17] Galkin S.P. (2004). Regulating radial-shear and screw rolling on the basis of the metal trajectory. Steel Transl..

[18] Galkin S.P. (2014). Radial shear rolling as an optimal technology for lean production. Steel Transl..

[19] Gordienko A.I., Pochivalov Y.I., Vlasov I.V., Mishin I.P. (2022). Structure formation and mechnical properties of low-carbon steel after lengthwise and cross rolling. Russ. Phys. J..

[20] Surikova N.S., Vlasov I.V., Derevyagina L.S., Gordienko A.I., Narkevich N.A. (2021). Influence of cross-screw rolling modes on mechanical properties and fracture toughness of pipe steel. Izvestiya. Ferrous Metallurgy.

[21] Skripalenko M.M., Romantsev B.A., Galkin S.P., Kaputkina L.M., Skripalenko M.N., Danilin A.V., Rogachev S.O. (2020). Forming features at screw rolling of austenitic stainless-steel billets. J. Materi. Eng. Perform..

[22] Panin S., Vlasov I., Moiseenko D., Maksimov P., Maruschak P., Yakovlev A., Gomorova J., Mishin I., Schmauder S. (2021). Increasing low-temperature toughness of 09Mn2Si steel through lamellar structuring by helical rolling. Metals.

[23] Panin S., Vlasov I., Maksimov P., Moiseenko D., Maruschak P., Yakovlev A., Schmauder S., Berto F. (2020). Increasing fatigue life of 09Mn2Si steel by helical rolling: Theoretical–experimental study on governing role of grain boundaries. Materials.

[24] Naizabekov A.B., Lezhnev S.N., Panin E.A. (2021). Formation of a gradient structure in austenitic stainless steel AISI 321 by radial-shear rolling. Solid State Phenom..

[25] Skripalenko M.M., Karpov B.V., Skripalenko M.N., Romantsev B.A., Galkin S.P., Kaputkina L.M., Yusupov V.S., Cheverikin V.V. (2021). Radial-shear rolling of titanium alloy billets with flat and profiled ends. Russ. Metall. Met..

[26] Xuan T.D., Sheremetyev V.A., Kudryashova A.A., Galkin S.P., Andreev V.A., Prokoshkin S.D., Brailovski V. (2020). Influence of the combined radial shear rolling and rotary forging on the deformation mode of the small-diameter rod billet made of titanium alloys. Russ. J. Non Ferr. Met..

[27] Stefanik A., Szota P., Mróz S., Wachowski M. (2022). Changes in the properties in bimodal Mg alloy bars obtained for various deformation patterns in the RSR rolling process. Materials.

[28] Dobatkin S., Galkin S., Estrin Y., Serebryany V., Diez M., Martynenko N., Lukyanova E., Perezhogin V. (2019). Grain refinement, texture, and mechanical properties of a magnesium alloy after radial-shear rolling. J. Alloy. Compd..

[29] Akopyan T.K., Gamin Y.V., Galkin S.P., Prosviryakov A.S., Aleshchenko A.S., Noshin M.A., Koshmin A.N., Fomin A.V. (2020). Radial-shear rolling of high-strength aluminum alloys: Finite element simulation and analysis of microstructure and mechanical properties. Mater. Sci. Eng. A.

[30] Arbuz A., Kawalek A., Ozhmegov K., Dyja H., Panin E., Lepsibayev A., Sultanbekov S., Shamenova R. (2020). Using of radial-shear rolling to improve the structure and radiation resistance of zirconium-based alloys. Materials.

[31] Gamin Y.V., Galkin S.P., Nguyen X.D., Akopyan T.K. (2022). Analysis of temperature-deformation conditions for rolling aluminum alloy Al–Mg–Sc based on FEM modeling. Russ. J. Non Ferr. Met..

[32] Sheremetyev V., Kudryashova A., Cheverikin V., Korotitskiy A., Galkin S., Prokoshkin S., Brailovski V. (2019). Hot radial shear rolling and rotary forging of metastable beta Ti-18Zr-14Nb (at. %) alloy for bone implants: Microstructure, texture and functional properties. J. Alloy. Compd..

[33] Gamin Y.V., Muñoz Bolaños J.A., Aleschenko A.S., Komissarov A.A., Bunits N.S., Nikolaev D.A., Fomin A.V., Cheverikin V.V. (2021). Influence of the radial-shear rolling (RSR) process on the microstructure, electrical conductivity and mechanical properties of a Cu–Ni–Cr–Si alloy. Mater. Sci. Eng. A.

[34] Bikmukhametova A., Galieva E., Valeev I., Klassman E., Musabirov I., Valitov V. (2021). The influence of radial shear rolling on the structure and properties of 58Ni-Cr-Mo-B-Al-Cu superalloy. Lett. Mater..

[35] Galkin S.P., Gamin Y.V., Kin T.Y. (2022). Analysis of temperature influence on strain–speed parameters of radial-shear rolling of Al-Zn-Mg-Ni-Fe alloy. Materials.

[36] Ünlü N. (2008). Preparation of high quality Al TEM specimens via a double-Jet electropolishing technique. Mater. Charact..

[37] Voort G.V., Geertruyden W.V., Dillon S., Manilova E. (2006). Metallographic preparation for electron backscattered diffraction. Microsc. Microanal..

[38] Panin E. ZIRCONIUM_Zr-1%Nb, 2020. Mendeley Data. https://data.mendeley.com/datasets/pg9wfwdxmz/1.

