# Supplementary figures and images for: Obtaining an Equiaxed Ultrafine-Grained State of the Longlength Bulk Zirconium Alloy Bars by Extralarge Shear Deformations with a Vortex Metal Flow
Source: Materials (Basel). 2023 Jan 25;16(3):1062. doi: 10.3390/ma16031062 (PMC9921253; doi:10.3390/ma16031062)

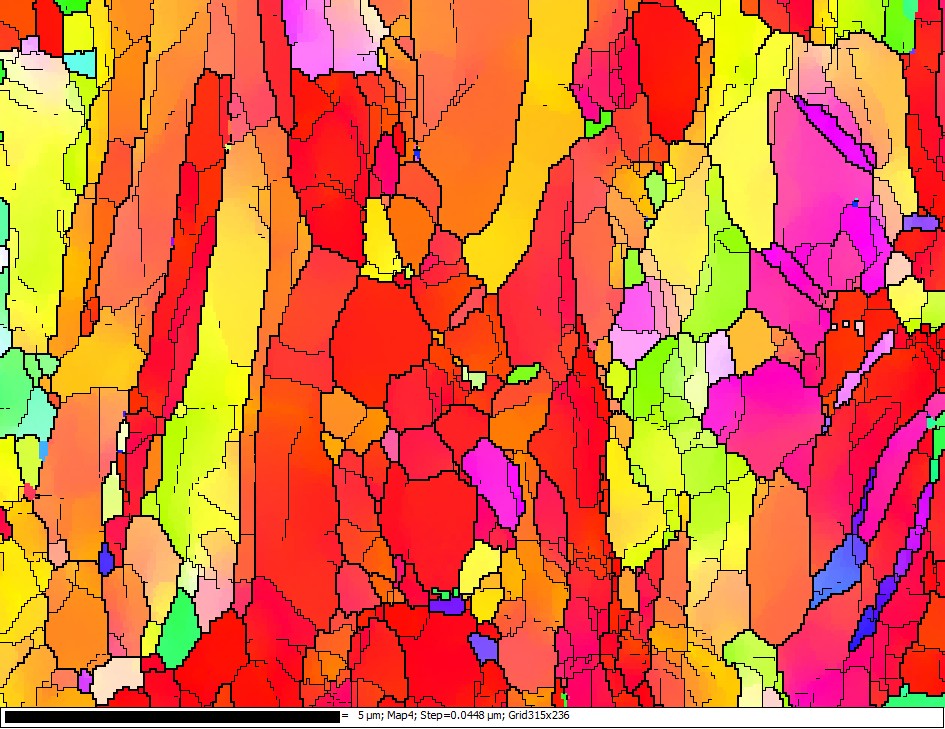

Supplement: Supplementary file 1 [file materials-16-01062-s001.zip › Figure S1 - 0 mm distance from the center.jpg]

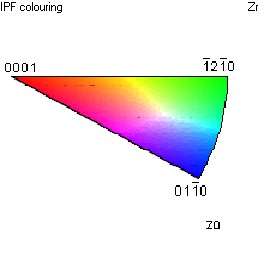

Supplement: Supplementary file 1 [file materials-16-01062-s001.zip › Figure S10 - EBSD legend IPF 1 colouring.jpg]

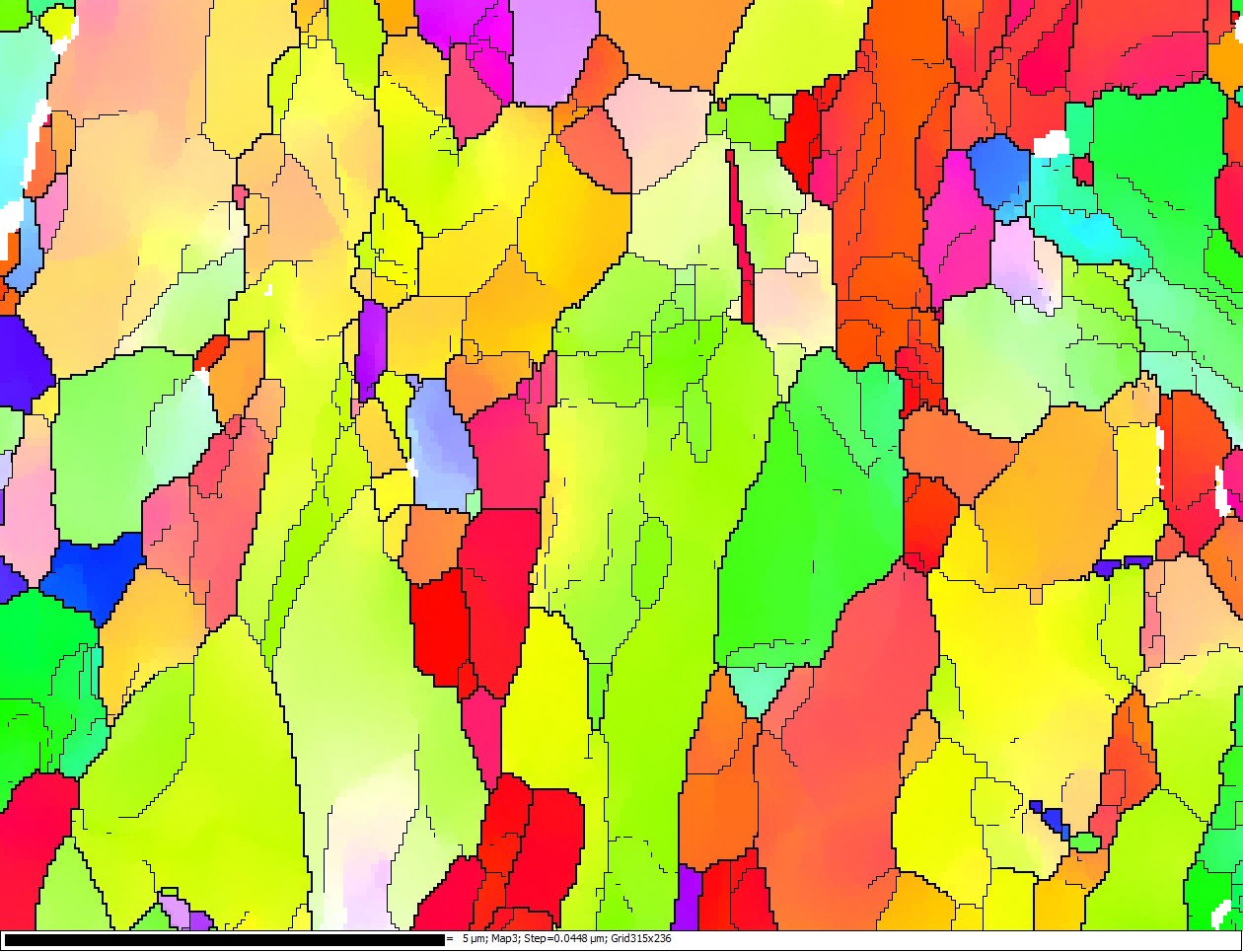

Supplement: Supplementary file 1 [file materials-16-01062-s001.zip › Figure S2 - 1 mm distance from the center.jpg]

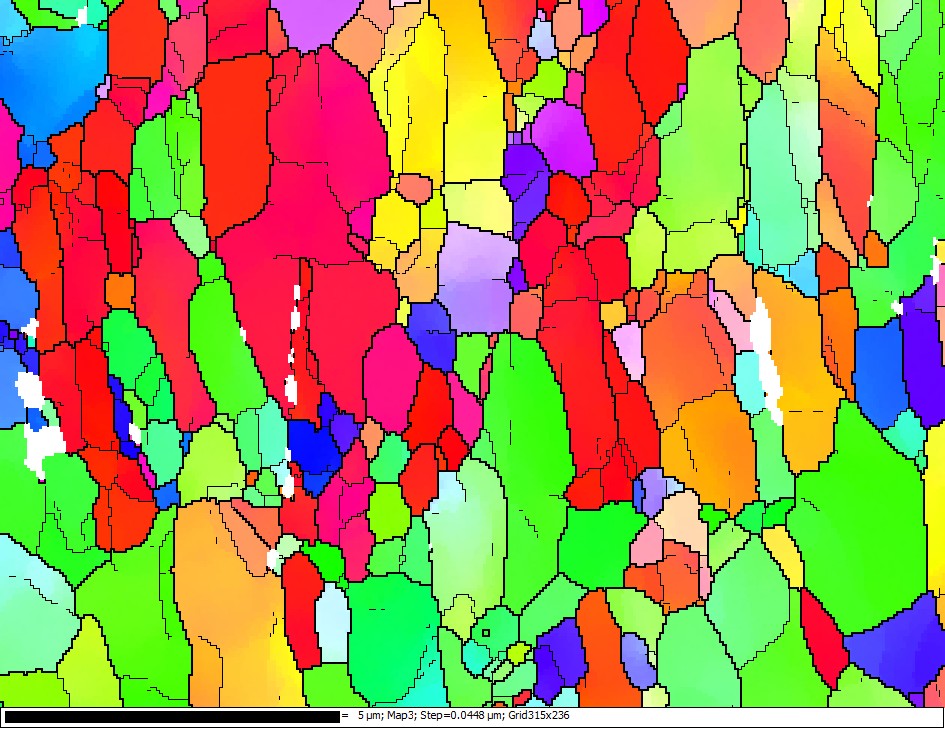

Supplement: Supplementary file 1 [file materials-16-01062-s001.zip › Figure S3 - 2 mm distance from the center.jpg]

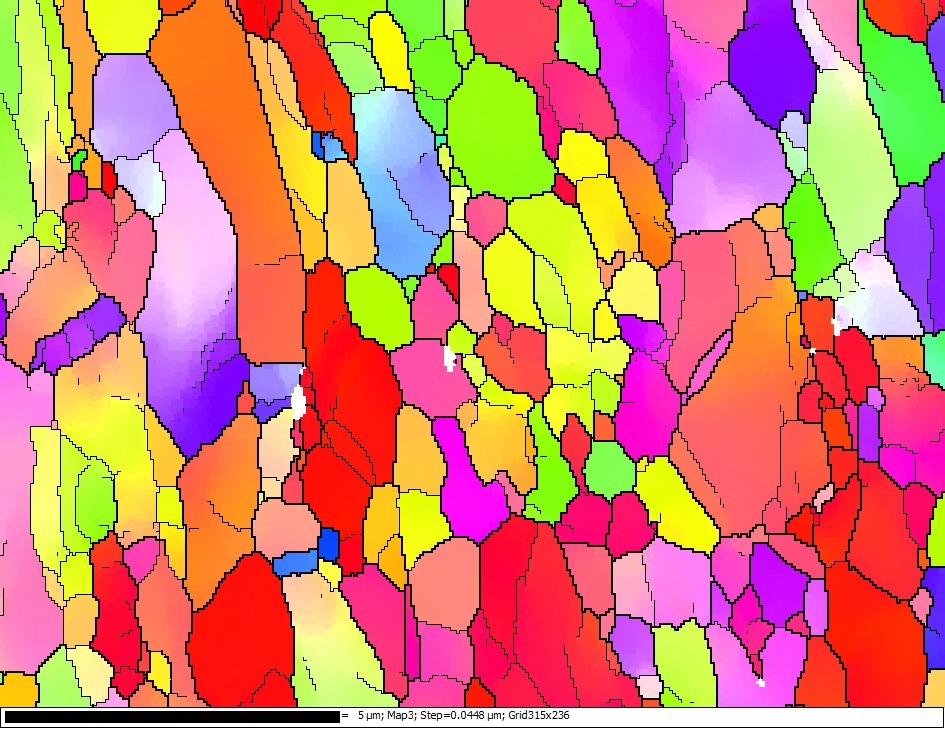

Supplement: Supplementary file 1 [file materials-16-01062-s001.zip › Figure S4 - 3 mm distance from the center.jpg]

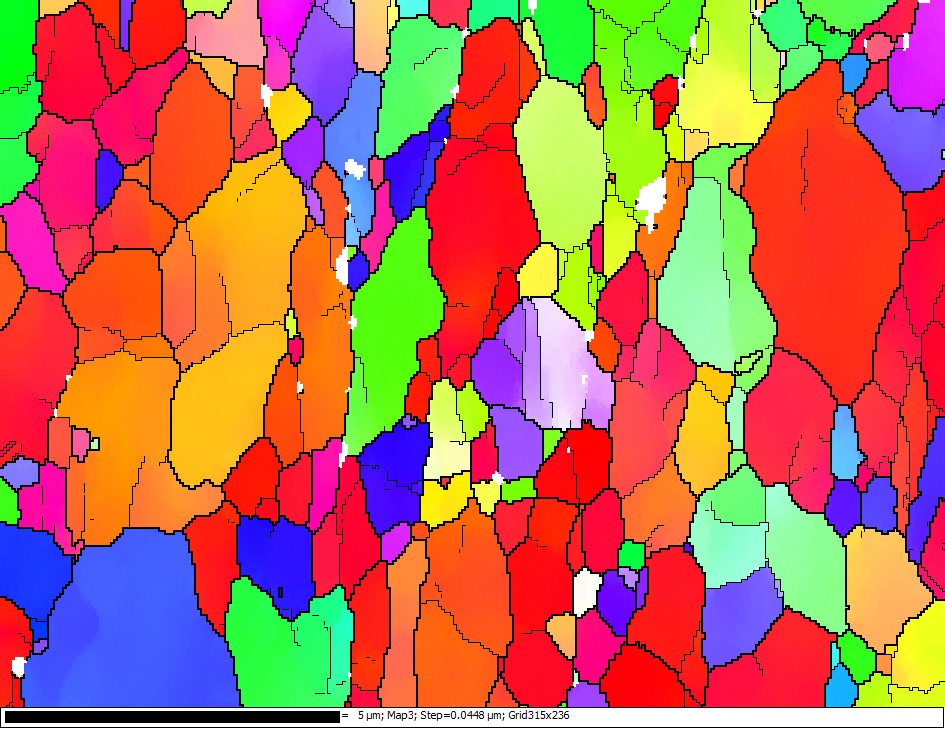

Supplement: Supplementary file 1 [file materials-16-01062-s001.zip › Figure S5 - 4 mm distance from the center.jpg]

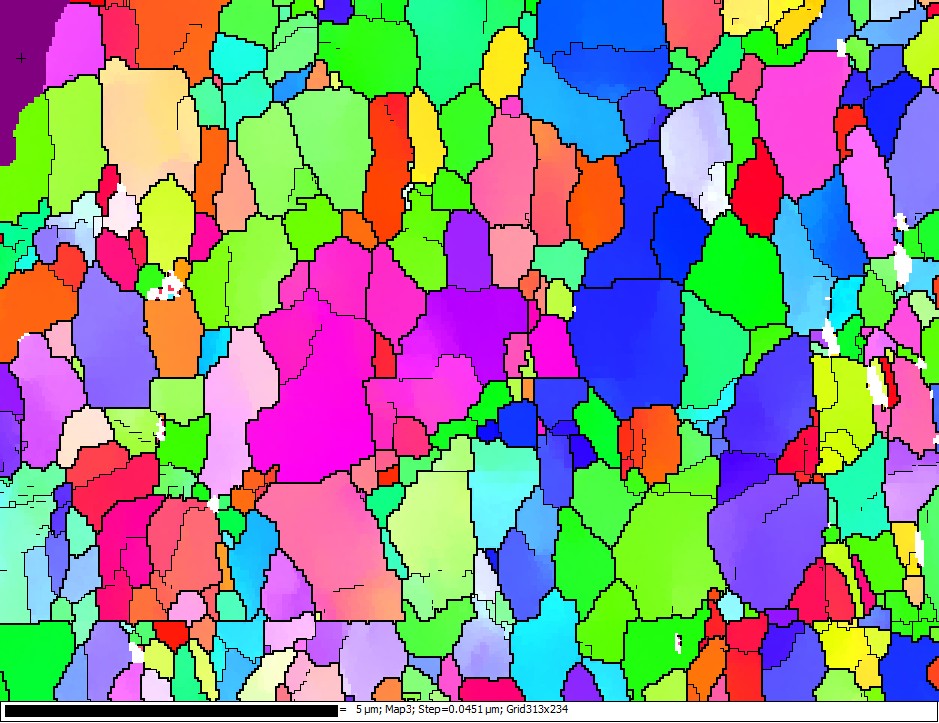

Supplement: Supplementary file 1 [file materials-16-01062-s001.zip › Figure S6 - 5 mm distance from the center.jpg]

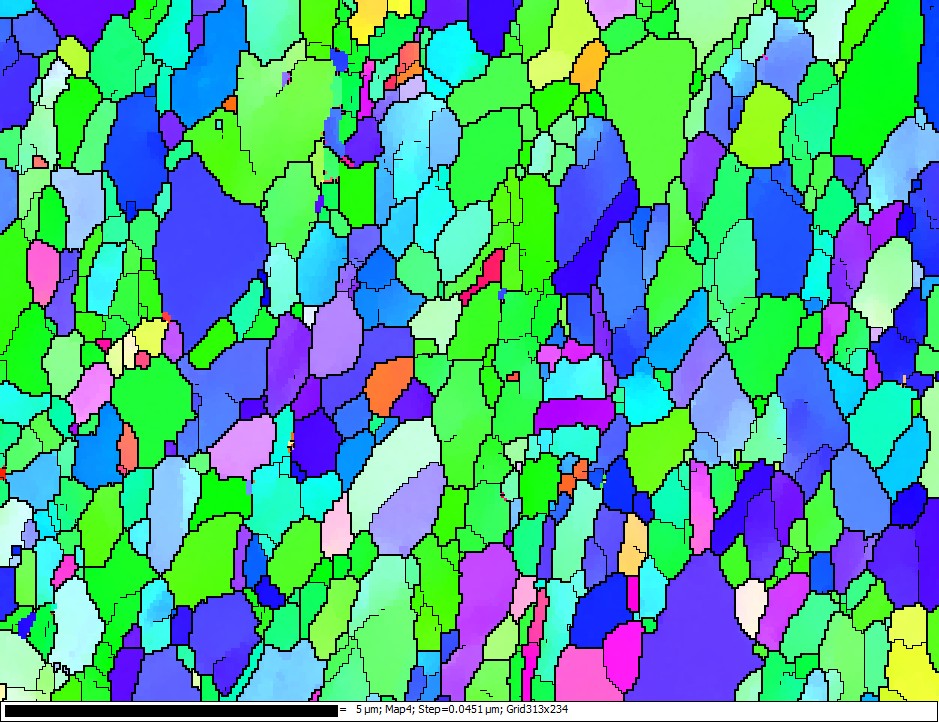

Supplement: Supplementary file 1 [file materials-16-01062-s001.zip › Figure S7 - 6 mm distance from the center.jpg]

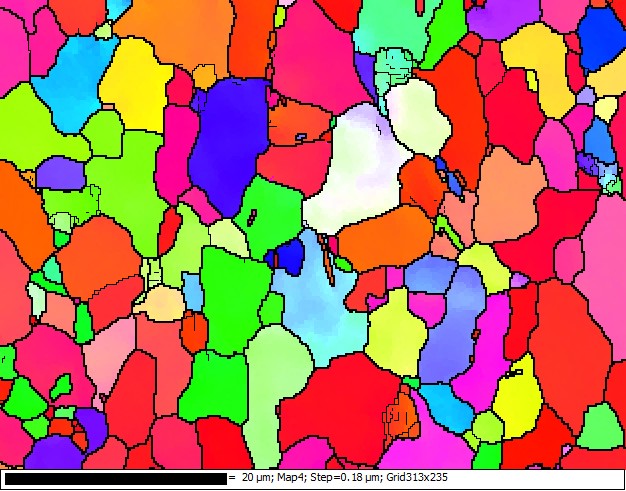

Supplement: Supplementary file 1 [file materials-16-01062-s001.zip › Figure S8 - INITIAL Structure by EBSD.jpg]

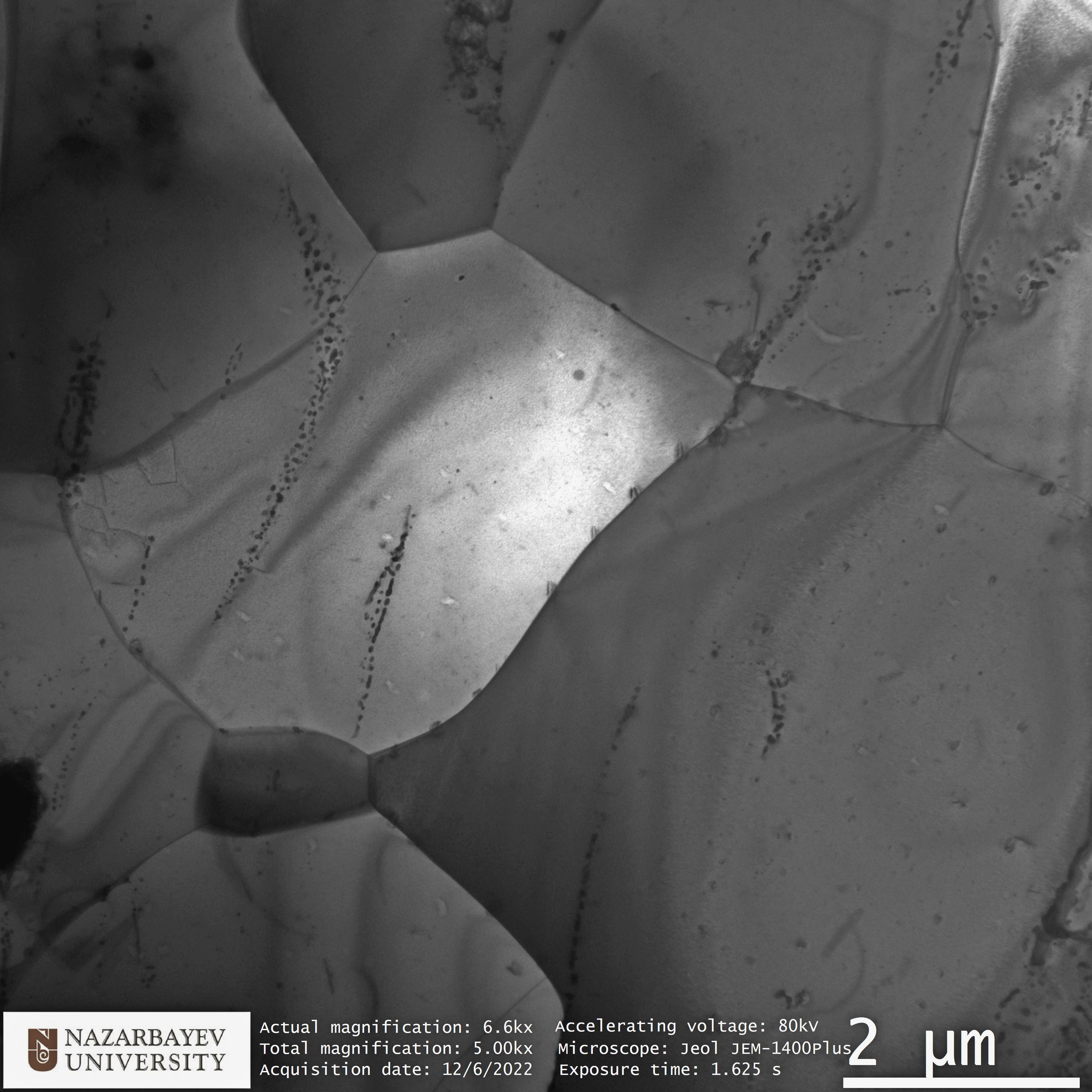

Supplement: Supplementary file 1 [file materials-16-01062-s001.zip › Figure S9 - INITIAL Structure by TEM.jpg]
